# Bactericidal Activity of Silver-Doped Chitosan Coatings via Electrophoretic Deposition on Ti_6_Al_4_V Additively Manufactured Substrates

**DOI:** 10.3390/polym15204130

**Published:** 2023-10-18

**Authors:** Arash Ghalayani Esfahani, Maria Sartori, Chiara Bregoli, Jacopo Fiocchi, Carlo Alberto Biffi, Ausonio Tuissi, Gianluca Giavaresi, Alessandro Presentato, Rosa Alduina, Angela De Luca, Alessia Cabrini, Cristina De Capitani, Milena Fini, Emanuele Gruppioni, Marino Lavorgna, Alfredo Ronca

**Affiliations:** 1 Institute for Polymers, Composites and Biomaterials (IPCB), National Research Council (Consiglio Nazionale delle Ricerche) (CNR), Via Gaetano Previati, 1/E, 23900 Lecco, Italy; alessia.cabrini@ipcb.cnr.it (A.C.); cristina.decapitani@cnr.it (C.D.C.); marino.lavorgna@cnr.it (M.L.); alfredo.ronca@cnr.it (A.R.); 2 Surgical Sciences and Technologies, IRCCS Istituto Ortopedico Rizzoli, Via Di Barbiano, 1/10, 40136 Bologna, Italy; maria.sartori@ior.it (M.S.); gianluca.giavaresi@ior.it (G.G.); angela.deluca@ior.it (A.D.L.); 3 Institute of Condensed Matter Chemistry and Technologies for Energy (ICMATE), National Research Council (Consiglio Nazionale delle Ricerche) (CNR), Via Gaetano Previati, 1/E, 23900 Lecco, Italy; chiara.bregoli@icmate.cnr.it (C.B.); jacopo.fiocchi@icmate.cnr.it (J.F.); carloalberto.biffi@cnr.it (C.A.B.); ausonio.tuissi@cnr.it (A.T.); 4 Department of Biological, Chemical and Pharmaceutical Sciences and Technologies (STEBICEF), University of Palermo, Viale delle Scienze, Bd. 16, 90128 Palermo, Italy; alessandro.presentato@unipa.it (A.P.); valeria.alduina@unipa.it (R.A.); 5 Scientific Directorate, IRCCS Istituto Ortopedico Rizzoli, Via Di Barbiano, 1/10, 40136 Bologna, Italy; milena.fini@ior.it; 6 INAIL Centro Protesi, Via Rabuina 14, Vigorso di Budrio, 40054 Bologna, Italy; e.gruppioni@inail.it

**Keywords:** chitosan, electrophoretic deposition, silver, postarthroplasty infection, antibacterial coating, additive manufacturing

## Abstract

Prosthetic reconstruction can serve as a feasible alternative, delivering both functional and aesthetic benefits to individuals with hand and finger injuries, frequent causes of emergency room visits. Implant-related infections pose significant challenges in arthroplasty and osteosynthesis procedures, contributing to surgical failures. As a potential solution to this challenge, this study developed a new class of silver (Ag)-doped chitosan (CS) coatings via electrophoretic deposition (EPD) on osseointegrated prostheses for infection therapy. These coatings were successfully applied to additively manufactured Ti_6_Al_4_V ELI samples. In the initial phase, the feasibility of the composite coating was assessed using the Thermogravimetric Analysis (TGA) and Attenuated Total Reflection (ATR) techniques. The optimized structures exhibited impressive water uptake in the range of 300–360%. Codeposition with an antibacterial agent proved effective, and scanning electron microscopy (SEM) was used to examine the coating morphology. Biologically, CS coatings demonstrated cytocompatibility when in direct contact with a fibroblast cell line (L929) after 72 h. When exposed to the *Staphylococcus epidermidis* strain (ATCC 12228), these coatings inhibited bacterial growth and biofilm formation within 24 h. These findings underscore the significant potential of this approach for various applications, including endoprostheses like hip implants, internal medical devices, and transcutaneous prostheses such as osseointegrated limb prosthetics for upper and lower extremities.

## 1. Introduction

Injuries to the hands and fingers are among the most frequent reasons for emergency room visits [1,2]. Reports indicate that the incidence in the United States (US) can reach as high as 45,000 amputations annually [3]. Furthermore, due to the finger’s crucial role in everyday activities and various professions, the consequences of an amputation can be absolutely devastating [4]. The recommended approach for repairing such injuries generally involves autologous reconstruction. However, in cases where autologous reconstruction is not an option, prosthetic reconstruction can serve as a feasible alternative, offering both functional and aesthetic benefits [5]. Infection stands out as the primary challenge when using osseointegrated transcutaneous prostheses, which have been designed to address upper- and lower-limb amputation cases [6,7,8,9,10]. Additionally, infection after joint replacement surgery is a serious and potentially life-threatening complication that can occur in patients who undergo surgical procedures for prosthetic implants. Moreover, infection induces the failure of prosthetic implantations, increasing costs for the healthcare system and resulting in high rates of morbidity and mortality. Despite the use of aseptic techniques and prophylactic antibiotics, infection can still occur nowadays in 1–2% of patients undergoing primary replacement surgery and up to 40% in the case of revision surgery [11], leading to increased morbidity, prolonged hospital stays, and higher healthcare costs. The primary approach for treating infections involves the administration of antibiotics, which, regrettably, can lead to several complications and disadvantages. Moreover, the distressing surge in antibiotic resistance is compounding these issues over time [12,13,14,15,16], as is the virulence of some bacterial strains that can develop biofilms on device surfaces, particularly bacteria belonging to the Staphylococcus genus. The biofilm produced by these bacteria protects them from the host defense system and bactericidal agents through several proposed mechanisms. Because bacteria in the biofilm are more resistant compared to their planktonic counterparts, the standard approach based on antibiotic treatments may not be sufficient to eradicate infection [17,18,19,20,21,22,23,24,25,26]. In this context, the use of new technologies has been investigated to reduce the incidence of postoperative infections [27,28].

To address this problem, the authors developed a new approach by creating a composite coating made of chitosan (CS) and silver (Ag) using the electrophoretic deposition (EPD) technique.

The EPD technique is a versatile and cost-effective method used to deposit thin films or coatings onto substrates with complex shapes (like orthopedic implants) and varying sizes. This method offers numerous advantages over traditional coating techniques, including high deposition efficiency, precise control over coating thickness, and the ability to deposit a wide range of materials [29].

Chitosan is a biopolymer that has garnered considerable attention in the field of biomedical applications. This is due to its various beneficial properties, including antibacterial, antifungal, and antitumor activities, as well as its capacity to promote tissue regeneration and wound healing [30]. These properties make it a promising material for various biomedical applications, such as drug delivery, tissue engineering, wound dressings, and implant coatings [31,32,33]. One of the most advantageous features of chitosan is its ability to chelate with a wide range of metal ions, particularly those belonging to the category of transition elements such as silver [34]. According to reports, CS–metal ion complexes exhibit significantly greater in vitro antibacterial activity when compared to both free CS and antimicrobial metal salts [33,34,35]. In addition, the choice of appropriate Therapeutic Metal Ions (TMIs) for chelation can support essential biological processes, including osteogenesis and angiogenesis. TMIs have the potential to interact with various biological structures and metabolic systems [36] and can elicit positive effects on tissue regeneration mechanisms by inhibiting the growth of prokaryotes while simultaneously interacting with specific mammalian cells [37].

Silver has long been recognized for its broad-spectrum antimicrobial effects, even against a wide range of multi-drug-resistant bacteria. Scientifically, it is established that silver exhibits biological activity when it exists in its ionic state as monoatomic Ag+, which can be dissolved in water-based environments [38]. Ionic silver compounds, such as silver nitrate and silver sulfadiazine, are often utilized for treating wounds [39].

So far, three distinct mechanisms of action for silver against microbes have been identified. The first mechanism involves the reaction of silver cations with the peptidoglycan component of bacterial cell walls, resulting in the formation of pores and puncturing the cell wall [40]. The second mechanism entails silver ions penetrating into bacterial cells, where they impede cellular respiration and disrupt metabolic pathways, leading to the production of reactive oxygen species [41]. The third mechanism involves silver disrupting DNA and the replication cycle of bacteria once it is within the cell [42].

The present study proposes the use of the EPD technique to create chitosan coatings on additively manufactured (AMed) substrates with two distinct levels of silver content. The primary objective is to develop a novel approach to prevent and/or inhibit the colonization of implant surfaces by microbial pathogens, which are known to cause postoperative infections by forming biofilms. Biological assessments have confirmed the antibacterial activity of the coating, validating the promising results of the EPD technique in creating Ag–chitosan coatings that can be successfully deposited on both standard and AMed prostheses. Such coatings offer advantages for both endoprostheses and transcutaneous prostheses. This antimicrobial tool represents an extremely promising technique that can provide a higher level of protection for implants.

## 2. Materials and Methods

### 2.1. Materials

Chitosan (CS) (deacetylated chitin, poly(D-glucosamine), medium molecular weight, Lot#BCCF3856) [43], L-Cysteine (for biochemistry, chelating agent, Lot#K53359238), acetic acid (99.8%, Lot#STBJ8845), Dulbecco’s phosphate-buffered saline (DPBS) (Lot#RNBG5989), and lysozyme from chicken egg white (Lot#SLBX2243) were all supplied by Sigma-Aldrich, St. Louis, MI, USA. Silver nitrate (99.8%, Lot#21L214109) [44], ethanol absolute (99.85%, Lot#22B174018), and water (CHROMASOLV^®^ Plus, for HPLC) were all supplied by VWR chemicals Avantor (Milan, Italy) and used without further purification. Merck provided Dulbecco’s Modified Eagle’s Medium (DMEM) (Lot#SLCK3894) and the Neutral Red-based assay (Lot#SLCL3641). GIBCO (Termo Fisher Scientific, Waltham, MA, USA) supplied penicillin/streptomycin (Lot#2441832), while Euroclone provided fetal bovine serum (Lot#EUS033883) and trypsin 0.05%/EDTA 0.02% (Lot#EUM00Z1). The Alamar Blue assay (Lot#2309199) came from Invitrogen, and the LDH assay kit (Lot#C110129) was provided by BioChain ((BioChain Institute Inc., Newark, CA, USA). Additionally, ChemCruz ((Santa Cruz Biotechnology Inc., Dallas, TX, USA) supplied the Neutral Red solution (Lot#I1916), and Invitrogen ((Termo Fisher Scientific, Waltham, MA, USA) provided Calcein-AM (Lot#L3224) for fluorescent staining. All the reagents used for the microbiological investigations were purchased from Merck Life Science S.r.l. in Milan, Italy.

### 2.2. Additively Manufactured Ti_6_Al_4_V ELI Substrate

Disk substrates (Ø = 10 mm, thickness 2 mm) were realized by means of laser powder bed fusion (LPBF) technology (mod. AM400 from Renishaw, Wotton-under-Edge, UK). Certified, medical-grade Ti_6_Al_4_V ELI powder was used to produce samples with optimized processing parameters (Table 1) [45].

Disk-shaped samples were realized along the plane perpendicular to the titanium building platform; indeed, the perpendicular surface (labeled as XZ surface) simulates well the surface of a prosthesis, which would usually be built along the perpendicular direction with respect to the platform. All parts were annealed at 850 °C for 1 h under high vacuum and then naturally cooled to remove residual stresses and obtain the desired microstructure [46]. AMed samples were cleaned by ultrasonic bath for 15 min, and XZ surfaces were polished with sandpapers up to #2500 grit and finally cleaned again in ultrasonic bath for 15 min.

### 2.3. Fabrication of CS/Ag Composite Coating

**Figure 1:**
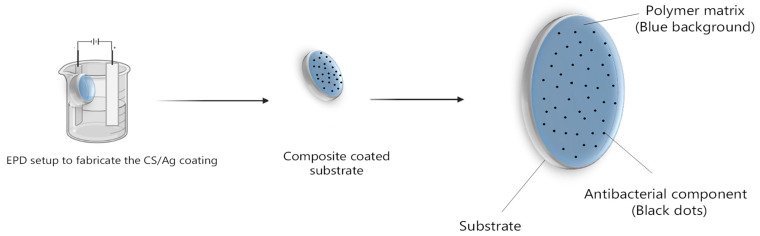
Schematic representation of CS/Ag coating fabricated using electrophoretic deposition (EPD).

Electrophoretic deposition bath was prepared by dissolving silver nitrate in 50% water + 50% ethanol bath (pH = 4.65, (CS) = 1 g L^−1^, (AgNO_3_) = 35 and 70 mg L^−1^) to produce Ag-loaded CS coatings [47]. Ti_6_Al_4_V ELI disk-shaped substrates were used as the cathode in an EPD cell; electrodes were positioned at a distance of 10 mm [48,49] in a lab-made EPD cell [50] (Figure 1). Processing conditions were optimized in order to achieve a uniform, homogeneous, and consistent deposition of coatings (V = 15 V, T = 10 min).

### 2.4. Chemico-Physical Characterization of the Coating

#### 2.4.1. Microstructural Characterization

Optimal conditions for uniform coating were defined by observing their morphology with a scanning electron microscope (SEM, mode. LEO 1430 from Zeiss, Oberkochen, Germany). Thereafter, the feasibility of the composite coating was assessed using Energy-dispersive X-ray spectroscopy (EDX; Oxford Instrument, Abingdon, UK); Thermogravimetric Analysis (TGA; Thermal Analysis System TGA 2 by Mettler-Toledo GmbH, Columbus, OH, USA) conducted on CS and Ag-loaded coatings in air, ranging from 27 °C to 800 °C (with a heating rate of 10 K min^−1^); and Attenuated Total Reflection (ATR; The Thermo Scientific™ Smart™ iTX, Walthan, MA, USA).

#### 2.4.2. Swelling Property

To assess the swelling properties of the coatings, they were immersed in phosphate buffer solution (PBS) at pH 7.4 and 37 °C. After obtaining the coatings, they were mechanically separated from the Ti_6_Al_4_V ELI substrates and dried. These dried coatings were then cut into small square specimens measuring 10 × 10 mm^2^, and their initial weight (W_d_) was recorded. At various time intervals (up to 72 h), the specimens were taken out of the solution and carefully dried using filter paper to remove any free water, retaining only the interstitial water trapped within the polymer network. The specimens were then weighed again (W_s_) and placed back into the solution; this process was repeated until equilibrium was reached [51]. At each time point, three specimens were analyzed, and the percentage of water uptake (WU) was calculated using the provided Equation (1):

(1) $$\mathrm{WU}\;(\%)=100\;[\frac{W_{s}-W_{d}}{W_{d}}]$$

where W_d_ is the dry weight, and W_s_ is the measured weight of the sample after swelling. 

#### 2.4.3. In Vitro Degradation

The degradation study involved immersing the coatings in phosphate-buffered saline (PBS) with a pH of 7.4 at 37 °C, containing 1.5 μg mL^−1^ lysozyme sourced from chicken egg white. The selection of this lysozyme concentration was based on its presence in human serum [52,53]. After obtaining the coatings, they were dried and cut into small circular specimens with a diameter of 7 mm, and their initial weight (dry weight) was recorded. Specimens were taken out of the solution at various time intervals (up to 3 days) and carefully dried at 37 °C in an oven overnight. Three specimens of each type of coating were examined at each time interval. The weight loss of the specimens was then measured to assess degradation.

(2) $$\mathrm{Weight}\;\mathrm{loss}\;(\%)=100\;[\frac{W_{t}-W_{o}}{W_{o}}]$$

where w_t_ is the weight of the dry specimen at different time points and w_o_ is the weight of the dry specimen before immersing in lysozyme solution. 

### 2.5. In Vitro Investigations

#### 2.5.1. Cytotoxicity Tests on Extract

All in vitro studies were performed on disk-shaped AMed Ti_6_Al_4_V ELI samples (Ø = 10 mm, height 2 mm), coated with chitosan only (CS), or with chitosan loaded with low Ag concentration (CS/LAg) or high Ag concentration (CS/HAg). Prior to in vitro tests, disks were steam-sterilized in an autoclave (Getinge Disinfectation AB-HS33 1p, Wayne, NJ, USA). Cytocompatibility of EPD structures was evaluated first by performing an in vitro cytotoxicity study in accordance with the International Guideline provided by UNI EN ISO 10993 Part 5 (2009) “Tests for in vitro cytotoxicity” [54].

Dulbecco’s Modified Eagle’s Medium supplemented with 10% fetal bovine serum and 100 IU/mL penicillin–100 μg/mL streptomycin was adopted as cell culture medium for the in vitro study. Murine fibroblast cell line (L929), preserved in −180 °C liquid nitrogen, was thawed for the test and incubated in a 75 mL flask in DMEM at 37 °C in a 5% CO_2_ humidified atmosphere. At 80% confluence, cells were treated with trypsin 0.05%/EDTA 0.02% (*w*/*v*) (Sigma, lot SLCC0835), released, counted, and resuspended at a concentration of 2.5 × 10^4^ cells/cm^2^ for the test. 

Six samples of Ti_6_Al_4_V ELI substrate coated with CS, CS/Lag, and CS/HAg were sterilely positioned in 6-well plates. Fibroblast L929 cell suspension was directly seeded in every well containing coated materials (onto and around each material), in six wells for negative control (CTR−) with DMEM only without any coated material, and in six wells for positive control (CTR+) in which 1% of phenol solution in DMEM was added to induce cell cytotoxic state. Plates with and without coated materials were incubated for 72 h at 37 °C in a 5% CO_2_ atmosphere. 

At the end of the experimental time, metabolic activity of fibroblast cells was measured by means of the Alamar Blue assay. The assay is based on the use of a non-toxic colorimetric indicator (resazurin), a non-fluorescent blue color that is turned into a fluorescent signal (resufurin) as a response to cellular metabolic activity. The fluorescence signal emitted by the cell’s cultures with coated materials and control conditions was quantified at 530ex–590em nm wavelengths using a microplate reader (VICTOR X2030, Perkin Elmer, Milano, Italy) and expressed as relative fluorescence units (RFUs). 

In addition to the Alamar Blue assay, the quantitative measure of intracellular enzyme Lactic Dehydrogenase (LDH) released in the culture medium was also performed according to the manufacturer’s instructions. The reaction product was measured with a spectrophotometer (iMARK, Biorad spectrophotometer, Hercules, CA, USA) at a wavelength of 490 nm. The measured values were then expressed in % of cytotoxicity by using the following equation:

(3) $$\%\;of\;Citotoxicity=\frac{\left(LDH\right)experimental\;coatings-\left(LDH\right)CTR\;negative}{\left(LDH\right)CTR\;positive-\left(LDH\right)CTR\;negative}$$

Fibroblasts grown around coated materials and in control conditions were stained with Neutral Red (NR) to qualitatively evaluate the overall morphology of the cells. Calcein, a fluorescein-based indicator, was used to directly visualize cells grown on the surface of the coated materials. Calcein-AM solution was incubated with chitosan coatings for 30 min at 37 °C. The cells labeled with both NR and Calcein staining were observed and imaged using an Eclipse Tj-U inverted microscope (Nikon Europe B.V., Amstelveen, The Netherlands), also equipped with a fluorescent setup with an excitation wavelength of 490 nm.

#### 2.5.2. Antibacterial Investigation

Gram-positive *S. epidermidis* (ATCC 12228) was adopted to test the antibacterial activity of pure chitosan and composite coatings of CS/Ag on Ti_6_Al_4_V ELI substrate. Firstly, bacterial cells were pre-cultivated for approximately 16 h at 37 °C with shacking (180 rpm) in Luria Bertani medium (hereafter named LB), containing (g L^−1^) sodium chloride (NaCl; 10), tryptone (10), and yeast extract (5). In cases where solidification of the LB medium was required, 15 g L^−1^ of bacteriological agar was added. After the pre-cultivation step, bacterial cells were inoculated into fresh LB medium at a microbial titer of 1 × 108 colony-forming units per milliliter of culture (CFU mL^−1^). Thus, 1 mL of inoculum was aliquoted into a multitier 24-well plate, where coatings were placed using sterile tweezers. Six specimens for each type of coating were considered.

The plate was subsequently incubated at 37 °C for 24 h under static conditions, allowing cell attachment to the scaffold and biofilm development. After the incubation step, planktonic cells were discarded, and each coating was thoroughly washed with sterile saline solution (0.9% *w*/*v* NaCl), removing loosely adherent cells from the scaffolds. The latter were placed in 15 mL sterile conical tubes containing 5 mL of saline solution, which were sonicated for 5 min at a frequency of 35 kHz using a sonicator bath to detach the bacterial cells from the scaffold surface. 

Finally, the number of CFU mL^−1^ featuring the biofilm formed on the coatings was evaluated by the spot plate count method after performing a decimal serial dilution of the sonication product and spotting 10 µL of each dilution onto LB agar recovery plates. The latter was incubated at 37 °C for 24 h under static conditions, allowing bacterial colony development. 

### 2.6. Statistical Data Analysis

The Statistical Package for the Social Sciences was used for the calculations (Minitab ExpressTM Version 1.4.0, Milan, Italy). The reported results are presented as mean ± standard deviation (SD). To determine significant differences between the two sets of data, a one-way ANOVA was conducted, followed by a Tukey post hoc test for pairwise comparisons. A value of *p* < 0.05 was considered statistically significant. The data derived from the biological investigations (metabolic activity, LDH quantification, and antibacterial activity) were analyzed using GraphPad Prism 9 (GraphPad Software, La Jolla, CA, USA). A one-way ANOVA test was used to analyze the statistical differences between the experimental conditions, followed by Sidak’s post hoc multiple comparison test. Data are reported as the average (n = 3) of the CFU mL^−1^ on a logarithmic scale with standard deviation (SD). 

## 3. Results

### 3.1. Physical and Chemical Properties of Deposited Coatings

#### 3.1.1. Morphology of the Composite Coating Fabricated by EPD

**Figure 2:**
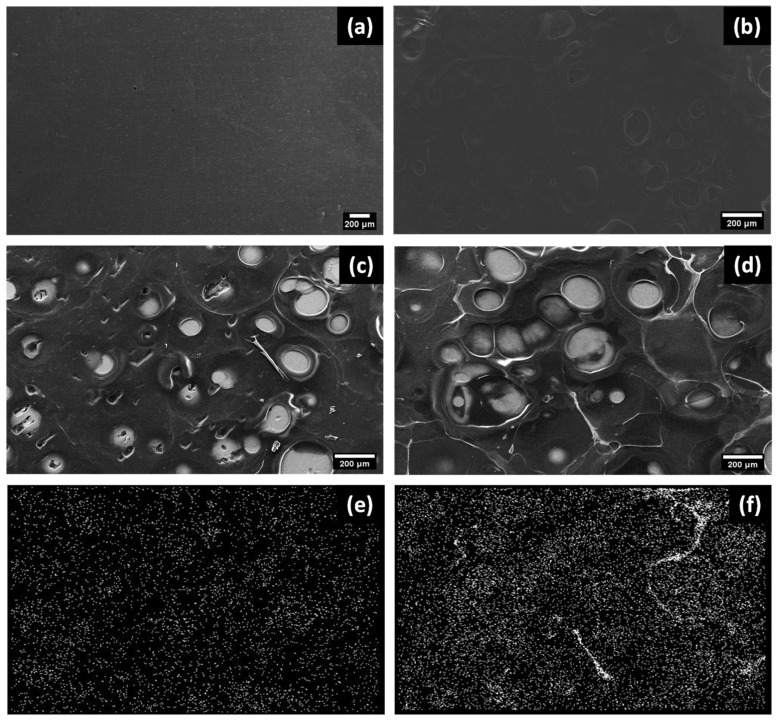
(**a**) SEM image of AMed Ti_6_Al_4_V ELI substrate; (**b**) SEM images of EPD of CS; (**c**) SEM images of EPD of CS/LAg composite coating with silver nitrate = 35 mg L^−1^; and (**d**) CS/HAg composite coating with silver nitrate = 70 mg L^−1^; (**e**,**f**) corresponding EDX maps showing local Ag concentration (scale bar: 200 µm).

The SEM images reported in Figure 2 show that the composite coatings obtained through EPD on AMed Ti_6_Al_4_V ELI substrates have a uniform and cohesive structure. When compared to pure CS coatings, the composite coatings exhibit a distinct morphology with a consistent distribution of bright spots. EDX maps support the viability of the composite coating and reveal the presence of Ag (Figure 2e,f).

The random porosity of the coatings on continuous substrates, as shown in Figure 2b, features pores ranging in size from 10 to 500 μm, as well as a secondary, random porosity with spherical pores measuring 10–100 μm in diameter. The electrolysis of water, which produces hydrogen gas, leads to the formation of a porous structure. The thickness of the resulting coating was found to be in the range of 125 ± 25 μm when in a dry state.

#### 3.1.2. Feasibility of EPD CS/Ag Composite Coating

**Figure 3:**
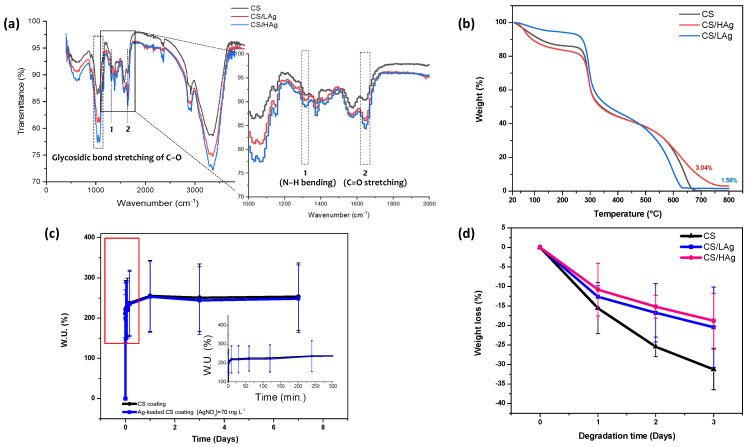
(**a**) Comparison between the ATR−FTIR spectra of pure chitosan and CS/Ag composite coatings, highlighting the main differences introduced by the silver doping; (**b**) TGA analysis (10 K min^−1^ heating rate) in air, ranging from 27 °C to 800 °C for chitosan and Ag−loaded coatings; (**c**) swelling behavior of CS and CS/Ag composite coatings. Tests were carried out over time in phosphate-buffered saline (PBS) at pH 7.4: (●) CS, (■) (Silver nitrate) = 70 mg L^−1^; (**d**) weight loss of CS and CS/Ag composite coatings immersed in PBS (pH 7.4) at 37 °C containing 1.5 μg mL^−1^ lysozyme: (▲) CS, (■) (Silver nitrate) = 35 mg L^−1^, (●) (Silver nitrate) = 70 mg L^−1^.

The results of the Energy-dispersive X-ray spectroscopy are reported in Table 2. The viability of the composite coating was further evaluated through the ATR and TGA techniques, as depicted in Figure 3a,b.

The presence of multiple minor peaks within the 900–1160 cm^−1^ range indicates the C–O–C stretching of the glycosidic bond linking the glucosamine monomers in CS. Furthermore, the peaks at 1650 cm^−1^ and 1320 cm^−1^, recognized as amide I and amide II, respectively, arise from the stretching of C=O and bending of N−H bonds.

Chelation with Ag ions brings about structural modifications in CS, leading to alterations in the relative transmittance of specific spectral bands involved in the creation of the Ag-CS complex. As observed in previous studies on complex formation, the intensities at 1320 cm^−1^ and 1650 cm^−1^ are increased due to C=O and N–H stretching vibrations, respectively, indicating the involvement of these groups in the reaction (Figure 3a). Additionally, the C–O peak at 1020 cm^−1^ exhibits a change in shape, possibly resulting from the steric effect of Ag cross-coordination with nearby CS chains, leading to an increase in glycosidic bond length. These findings provide confirmation of the successful formation of Ag-CS complex coatings during the electrodeposition process.

The presence of silver in the coatings was verified through TGA. The initial thermogravimetric peak in Figure 3b, at around 80 °C and a corresponding 10% weight loss, is attributed to the evaporation of water adsorbed or connected to the chitosan chains through weak hydrogen bonds. The decomposition temperature (TD) of the series of coatings was similar to that of chitosan (TD = 313 °C) [55,56]. An increase in residual weight was observed with an increase in Ag concentration, leading to a 68.18 ± 0.06% yield of Ag loading.

#### 3.1.3. Swelling

The swelling behavior of the chitosan and composite coatings up to 7 days in PBS is depicted in Figure 3c. Each of the specimens demonstrated significant water uptake (W.U. %) within the initial 10 min, after which the curves leveled off, reaching a plateau value. Furthermore, the presence of Ag did not significantly alter the swelling behavior of the CS coating. This characteristic behavior of CS makes it a favorable matrix for the controlled release of pharmaceuticals [57] and antibacterial agents such as silver.

#### 3.1.4. In Vitro Degradation

In Figure 3d, the in vitro degradation results are summarized. Over time, there was a noticeable increase in weight loss for both the CS and CS/Ag composite coatings. The CS sample exhibited 31.2% degradation after 3 days, while the CS/LAg and CS/HAg composite structures showed 20.42% and 18.8% degradation, respectively.

### 3.2. Cytotoxicity Assessment and Bactericidal Activity

#### 3.2.1. Cytotoxicity Test with a Direct Contact Approach

**Figure 4:**
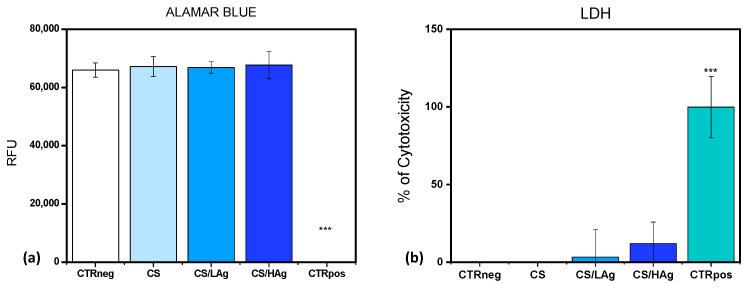
(**a**) Histogram related to the viability results obtained with Alamar Blue test; (**b**) Histogram of the values obtained for the quantification of LDH enzyme in culture supernatant at 72 h. (***: CTRpos vs. all, *p* < 0.0005).

The initial series of biological tests, aimed at assessing the safety of novel materials, scaffolds, coatings, or devices intended for human tissue applications, typically relies on methodologies recommended by the International Agency, as delineated in “UNI EN ISO 10993:5-2009 Biological evaluation of medical devices—Tests for in vitro cytotoxicity”. In accordance with this standard, we conducted an investigation into chitosan coatings by evaluating metabolic activity and cellular membrane integrity following direct exposure to a representative fibroblast cell line for a duration of 72 h, adhering to UNI EN ISO protocols. Fibroblasts were selected due to their significance as a fundamental component of soft tissue, playing a pivotal role in the processes of wound healing and skin regeneration. Notably, the metabolic activity of cells cultivated in direct contact with these coatings, including pure chitosan and chitosan variants with differing silver content (low and high), exhibited values comparable to those of the negative control (CTRneg). This control group consisted of fibroblasts cultivated on a plastic substrate, and the results indicated an absence of cytotoxic reactions (Figure 4a). This result was substantiated through LDH quantification, with LDH serving as an enzyme released into the culture medium in response to cellular membrane damage, thereby functioning as a biomarker for cytotoxicity and cytolysis. Consistent with the observations regarding metabolic activity, the percentages of cytotoxicity exhibited no significant differences among the different chitosan coatings and the CTRneg control group (Figure 4b). The results derived from the pure CS coatings align with the existing literature regarding the biocompatibility of chitosan polymers. Likewise, the composite coatings with silver obtained through EPD exhibit equivalent biocompatibility, with results that are in harmony with these findings.

**Figure 5:**
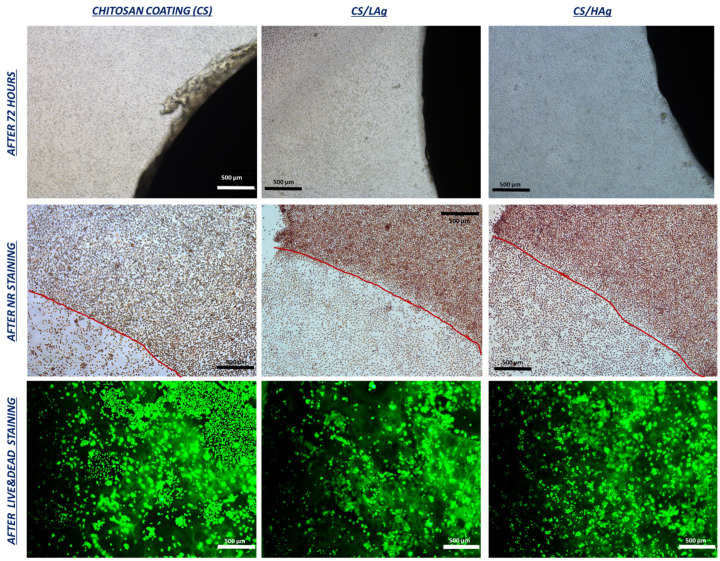
(Upper row) The fibroblast cultures with different coatings at the end of the experimental time of 72 h, prior to execution of the different investigations. (Middle row) Representative images acquired after Neutral Red staining, where the red line marks the point of transition between cells grown around the coating and those grown beneath the coating, providing further confirmation of cytocompatibility. (Bottom row) Fluorescence images that demonstrate the viability of cells grown in direct contact with the coating’s surfaces. All images were captured with the optical microscope Eclipse Tj-U inverted microscope (NIKON), also equipped with a fluorescent setup at 4× magnification (scale bar: 500 µm).

Neutral Red (NR) is a non-toxic vital dye that accumulates within lysosomes via an active transport mechanism, contingent upon the integrity of cellular membranes. This staining assay provides qualitative insights into cell morphology, membrane integrity, and, by extension, cell viability. It serves as a supplementary metric for evaluating the potential cytotoxicity of coatings, particularly those containing silver. In Figure 5, a representative qualitative panel illustrates the overall appearance of the cell culture at the end of the experimental period before conducting the test (upper row in Figure 5). After staining with NR, fibroblast morphology appears comparable in samples with silver coatings when compared to pure chitosan coatings (middle row in Figure 5). Finally, in Figure 5, the bottom images demonstrate that cells grew in direct contact with the materials. This was observed through fluorescent Calcein staining, allowing for the visualization of viable cells on the material surfaces. Employing various visualization techniques (bright field, Neutral Red, and Calcein staining), no significant differences in cell morphology or density between chitosan and chitosan combined with silver were found. Cells exhibited a spindle-like shape with well-defined cell membranes, even when in direct contact with the edges of the coatings.

#### 3.2.2. Antibacterial Investigation

**Figure 6:**
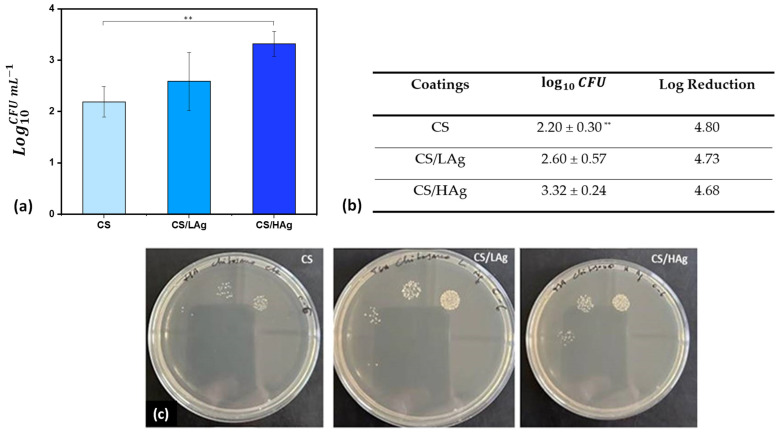
Microbiological results of coating composed of chitosan (CS) and chitosan combined with different contents of silver (CS/Lag or CS/Hag) against *S. epidermidis* bacterial strain, represented as a histogram (**a**) or reported in a table with “Log Reduction” in comparison to 10^8^ CFU/mL original inoculum (**b**). Subfigure (**c**) displays representative images of Petri dishes with serial dilutions obtained using the spread plate method. (**: CS vs. CS/HAg, *p* < 0.005).

The bactericidal activity of the coatings in preventing biofilm growth was evaluated against a relevant pathogenic gram-positive strain commonly found in infected wounds, represented by *S. epidermidis*. This bacterium, once considered a harmless commensal, has emerged as a significant opportunistic pathogen responsible for nosocomial infections, including joint prosthesis-related infections. The mechanism of infection is believed to involve the direct inoculation of skin-colonizing strains during surgery. *S. epidermidis* infections are challenging to treat due to their ability to evade the immune system and antibiotic therapy. Two mechanisms, bacterial invasion of non-professional phagocytes like osteoblasts and biofilm formation, have been proposed to be involved in orthopedic device infections [58]. Figure 6 shows the results of the adhesion of *S. epidermidis* to the various coatings. The viable bacteria after 24 h averaged 2.20 ± 0.30 for pure chitosan coating, 2.60 ± 0.57 for chitosan with low silver content, and 3.32 ± 0.24 for chitosan with high silver content. Consequently, the amounts of *S. epidermidis* that adhered to the coating surfaces were significantly lower than the initial inoculation levels (×10^8^/mL).

In accordance with our perspective, chitosan was utilized since prior research has shown that it contains bioactive groups like hydroxyl and amino groups. These groups demonstrate antimicrobial effects by interacting with the negatively charged cell membranes of bacteria. Chitosan disrupts bacterial functions, hindering nutrient transport, causing metabolic disruptions, and inhibiting bacterial growth and reproduction, aligning with our perspective [59].

## 4. Discussion

After fabricating the CS/Ag composite coating on AMed substrates using EPD, the feasibility was confirmed using the X-ray mapping and EDX spectrum techniques. The hydrolysis of water during the EPD process resulted in the formation of a porous structure in the pure CS material. Ag was predominantly observed along the borders of these pores, likely due to the higher current density in that particular area [29,60,61]. EDX and TGA analyses conducted on CS/Ag coatings demonstrated the presence of silver (Table 2). These results emphasize the possibility of silver being physically attached to CS macromolecules, possibly through weak bonds or trapped within the CS macromolecular structure. The degradation of CS has already been thoroughly studied [62,63,64,65]. The behavior of swelling in both CS and CS/Ag composite coatings indicates their suitability as effective matrices for the controlled release of antibacterial agents. 

Generally, polysaccharides undergo enzymatic hydrolysis for degradation. It is well established that lysozyme enzymatically depolymerizes CS in human serum [63]. The enzyme hydrolyzes the glycosidic bonds of polysaccharides, leading to biodegradation. Lysozyme contains a hexameric binding site [60], and hexasaccharide chains containing acetylated units initiate the initial degradation of the CS and CS/Ag composite coatings [66]. According to research, the optimal time to prevent biofilm formation following implantation surgery is within the first 6 h [65]. However, some types of bacteria can still form biofilms at the implant–tissue interface, even over a longer period of time [28,67,68]. According to the swelling behavior (Figure 3c), there is a rapid release of Ag within the first hour. Moreover, substantial weight loss in CS was only observed after prolonged incubation in PBS [69]. Additionally, in the presence of bacteria, it is generally observed that pH decreases [70]. This leads to the protonation of the amide groups on CS, resulting in the formation of the hydrophilic NH_3_^+^ group. Consequently, there is an electrostatic repulsion between the protonated amino groups, which weakens both the intermolecular and intramolecular hydrogen bonding interactions among CS molecules. As a result, the buffer solution can easily diffuse into the network, leading to an increase in the equilibrium swelling ratios. This increased swelling facilitates a faster diffusion rate from the matrix to the exterior. Consequently, the agents embedded within the matrix are released more easily and rapidly [71]. 

When assessing the bactericidal activity of CS/Ag coatings, several factors need to be considered. Firstly, CS inherently exhibits notable antibacterial properties against a wide range of bacteria. This effect is attributed to the interaction between the positively charged CS and the negatively charged microbial cell wall, which subsequently results in the leakage of intracellular components [72]. Another important aspect to consider is the pH-responsive swelling behavior of CS, making it an appropriate matrix for the controlled release of pharmaceuticals. As described in the introduction, three recognized mechanisms elucidate how silver exerts its antimicrobial effects on microbes, which allows it to exhibit potential effectiveness against both planktonic and biofilm cells. When silver is combined with chitosan, silver ions can bind to the functional groups, forming stable coordination complexes between chitosan and silver ions [73]. This combination should reduce the cytotoxicity of silver and further enhance its antibacterial properties, as chitosan can also serve as a stabilizing agent for silver, facilitating its release over time and its interaction with bacteria.

Cytotoxicity tests have indeed confirmed the compatibility of the chitosan coatings with both low and high silver contents. The microbiological findings reveal a significant decrease in bacterial load—over 4 logarithmic units—when compared to the initial inoculum. This underscores their suitability for application on implant surfaces. From a microbiological perspective, a greater antibacterial efficacy was expected in the composite coatings, as the boosting effect conferred by silver could be hypothesized.

## 5. Conclusions

The addition of silver can enhance the antimicrobial characteristics of chitosan, thereby boosting its ability to prevent and control microbial activity more effectively. Furthermore, chitosan plays a crucial role as a stabilizing agent, and this dual function not only taps into the antimicrobial potential of silver but also enhances its stability and bioactivity. 

The present results, obtained using the electrophoretic deposition (EPD) technique, demonstrate that chitosan can be effectively combined with silver, resulting in enhanced polymer stability as indicated by in vitro degradation tests. Furthermore, there were no signs of cytotoxicity, especially in the case of composite coatings. 

The implementation of similar coatings holds great promise for two key objectives. Firstly, it aims to reduce infections by over 4 logarithmic units in medical devices such as endoprostheses, including hip stems, and osseointegrated transcutaneous prostheses, where skin tissue infections can lead to significant complications. Secondly, it addresses the substantial challenge of achieving a uniform and durable coating on complex-shaped prosthetic devices.

By further optimizing the silver and chitosan combination, a new tool may be developed that not only addresses microbial infections but also combats bacterial resistance and contributes to advancing knowledge in the field of infection prevention.

Taken together, additional research, such as an in vivo animal model, is required to provide important information about the efficacy of pre-clinical preventative or therapeutic modalities against implant-related infections. This preliminary investigation is essential before conducting more extensive studies on human subjects.

## Figures and Tables

**Table 1:** Process parameters used for printing Ti_6_Al_4_V ELI substrates.

Process Parameters	Value
Power (W)	200
Exposure time (µs)	50
Layer thickness (µm)	30
Point distance (µm)	75
Hatch distance (µm)	65
Atmosphere	argon
Strategy	Meander

**Table 2:** Chemical composition of CS/Ag composite coatings measured by EDX. CS/LAg: (silver nitrate) = 35 mg L^−1^; and CS/HAg: (silver nitrate) = 70 mg L^−1^.

Sample	Weight (%)				
	C	O	Al	Ti	Ag
CS/LAg	40.7	38.28	0.84	18.91	1.27
CS/HAg	38.5	38.52	0.88	19.99	2.11

## Data Availability

The data presented in this study are available on request from the corresponding author. The data are not publicly available due to privacy restrictions.

## References

[1] Conn J.M., Annest J.L., Ryan G.W., Budnitz D.S. (2005). Non-work-related finger amputations in the United States, 2001–2002. Ann. Emerg. Med..

[2] Mahmoudi E., Swiatek P.R., Chung K.C., Ayanian J.Z. (2016). Racial Variation in Treatment of Traumatic Finger/Thumb Amputation: A National Comparative Study of Replantation and Revision Amputation. Plast. Reconstr. Surg..

[3] Reid D.B.C., Shah K.N., Eltorai A.E.M., Got C.C., Daniels A.H. (2019). Epidemiology of Finger Amputations in the United States from 1997 to 2016. J. Hand Surg. Glob. Online.

[4] Peterson S.L., Peterson E.L., Wheatley M.J. (2014). Management of Fingertip Amputations. J. Hand Surg. Am..

[5] Manrique O.J., Ciudad P., Doscher M., Torto F.L., Liebling R., Galan R. (2017). Osseointegrated finger prostheses using a tripod titanium mini-plate. Arch. Plast. Surg..

[6] Hebert J.S., Rehani M., Stiegelmar R. (2017). Osseointegration for Lower-Limb Amputation. JBJS Rev..

[7] Al Muderis M., Lu W., Li J.J. (2017). Osseointegrated Prosthetic Limb for the treatment of lower limb amputations. Unfallchirurg.

[8] Hoyt B.W., Walsh S.A., Forsberg J.A. (2020). Osseointegrated prostheses for the rehabilitation of amputees (OPRA): Results and clinical perspective. Expert Rev. Med. Devices.

[9] Bregoli C., Biffi C.A., Morellato K., Gruppioni E., Primavera M., Rampoldi M., Lando M., Adani R., Tuissi A. (2022). Osseointegrated Metallic Implants for Finger Amputees: A Review of the Literature. Orthop. Surg..

[10] Bregoli C., Stacchiotti F., Fiocchi J., Ferrari R., Biffi C.A., Morellato K., Gruppioni E., Tuissi A. (2023). A biomechanical study of osseointegrated patient-matched additively manufactured implant for treatment of thumb amputees. Med. Eng. Phys..

[11] Gatti M., Barnini S., Guarracino F., Parisio E.M., Spinicci M., Viaggi B., D’Arienzo S., Forni S., Galano A., Gemmi F. (2022). Orthopaedic Implant-Associated Staphylococcal Infections: A Critical Reappraisal of Unmet Clinical Needs Associated with the Implementation of the Best Antibiotic Choice. Antibiotics.

[12] Darouiche R.O. (2004). Treatment of Infections Associated with Surgical Implants. N. Engl. J. Med..

[13] Zimmerli W., Trampuz A., Ochsner P.E. (2004). Prosthetic-Joint Infections. N. Engl. J. Med..

[14] Trampuz A., Widmer A.F. (2006). Electrophoretic deposition of hydroxyapatite. Curr. Opin. Infect. Dis..

[15] Pulido L., Ghanem E., Joshi A., Purtill J.J., Parvizi J. (2008). Periprosthetic Joint Infection: The Incidence, Timing, and Predisposing Factors. Clin. Orthop. Relat. Res..

[16] Del Pozo J.L., Patel R. (2009). Infection Associated with Prosthetic Joints. N. Engl. J. Med..

[17] Høiby N., Bjarnsholt T., Givskov M., Molin S., Ciofu O. (2010). Antibiotic resistance of bacterial biofilms. Int. J. Antimicrob. Agents.

[18] Lindsay D., von Holy A. (2006). Bacterial biofilms within the clinical setting: What healthcare professionals should know. J. Hosp. Infect..

[19] Stewart P.S., Costerton J.W. (2001). Antibiotic resistance of bacteria in biofilms. Lancet.

[20] Zhao L., Chu P.K., Zhang Y., Wu Z. (2009). Antibacterial coatings on titanium implants. J. Biomed. Mater. Res.—Part B Appl. Biomater..

[21] Jiranek W.A., Hanssen A.D., Greenwald A.S. (2006). Antibiotic-Loaded Bone Cement for Infection Prophylaxis in Total Joint Replacement. J. Bone Jt. Surg..

[22] Cui Q., Mihalko W.M., Shields J.S., Ries M., Saleh K.J. (2007). Antibiotic-Impregnated Cement Spacers for the Treatment of Infection Associated with Total Hip or Knee Arthroplasty. JBJS.

[23] Mittal Y., Fehring T.K., Hanssen A., Marculescu C., Odum S.M., Osmon D. (2007). Two-Stage Reimplantation for Periprosthetic Knee Infection Involving Resistant Organisms. J. Bone Jt. Surg..

[24] Diwanji S.R., Kong I.K., Park Y.H., Cho S.G., Song E.K., Yoon T.R. (2008). Two-stage reconstruction of infected hip joints. J. Arthroplast..

[25] Chiu F.-Y., Lin C.-F.J. (2009). Antibiotic-Impregnated Cement in Revision Total Knee Arthroplasty. J. Bone Jt. Surgery-American Vol..

[26] Toulson C., Walcott-Sapp S., Hur J., Salvati E., Bostrom M., Brause B., Westrich G.H. (2009). Treatment of infected total hip arthroplasty with a 2-stage reimplantation protocol: Update on &quot;our institution’s&quot; experience from 1989 to 2003. J. Arthroplast..

[27] Campoccia D., Montanaro L., Arciola C.R. (2006). The significance of infection related to orthopedic devices and issues of antibiotic resistance. Biomaterials.

[28] Hetrick E.M., Schoenfisch M.H. (2006). Reducing implant-related infections: Active release strategies. Chem. Soc. Rev..

[29] Besra L., Liu M. (2007). A review on fundamentals and applications of electrophoretic deposition (EPD). Prog. Mater. Sci..

[30] Cabrini A., Esfahani A.G., Petraconi A., Lavorgna M., De Nardo L., Buonocore G.G., Andrade R.J.E., Cerruti P. (2023). Progress in Organic Coatings Ultrasonic spray deposition of PEGDE-crosslinked chitosan/graphene oxide coatings for enhancing gas barrier properties of polybutylene succinate films. Prog. Org. Coat..

[31] Simchi A., Pishbin F., Boccaccini A.R. (2009). Electrophoretic deposition of chitosan. Mater. Lett..

[32] Malafaya P.B., Silva G.A., Reis R.L. (2007). Natural-origin polymers as carriers and scaffolds for biomolecules and cell delivery in tissue engineering applications. Adv. Drug Deliv. Rev..

[33] Varoni E.M., Altomare L., Cochis A., GhalayaniEsfahani A., Cigada A., Rimondini L., De Nardo L. (2016). Hierarchic micro-patterned porous scaffolds via electrochemical replica-deposition enhance neo-vascularization. Biomed. Mater..

[34] Gritsch L., Lovell C., Goldmann W.H., Boccaccini A.R. (2018). Fabrication and characterization of copper(II)-chitosan complexes as antibiotic-free antibacterial biomaterial. Carbohydr. Polym..

[35] Wang X., Du Y., Fan L., Liu H., Hu Y. (2005). Chitosan- metal complexes as antimicrobial agent: Synthesis, characterization and Structure-activity study. Polym. Bull..

[36] Xue B., Li Q., Wang L., Deng M., Zhou H., Li N., Tan M., Hao D., Du H., Wang Q. (2023). Ferric-ellagate complex: A promising multifunctional photocatalyst. Chemosphere.

[37] Mouriño V., Cattalini J.P., Boccaccini A.R. (2012). Metallic ions as therapeutic agents in tissue engineering scaffolds: An overview of their biological applications and strategies for new developments. J. R. Soc. Interface.

[38] Hoffman R.K., Surkiewicz B.F., Chambers L.A., Phillips C.R. (1953). Bactericidal Action of Movidyn. Ind. Eng. Chem..

[39] Fong J., Wood F. (2006). Nanocrystalline silver dressings in wound management: A review. Int. J. Nanomed..

[40] Jung W.K., Koo H.C., Kim K.W., Shin S., Kim S.H., Park Y.H. (2008). Antibacterial Activity and Mechanism of Action of the Silver Ion in *Staphylococcus aureus* and *Escherichia coli*. Appl. Environ. Microbiol..

[41] Morones-Ramirez J.R., Winkler J.A., Spina C.S., Collins J.J. (2013). Silver enhances antibiotic activity against gram-negative bacteria. Sci. Transl. Med..

[42] Patton M.Q. (1980). Kinetic studies of the interaction between silver ion and deoxyribonucleic acid. Chem. Lett..

[43] Kim H.S., Lee S.H., Eun C.J., Yoo J., Seo Y.S. (2020). Dispersion of chitosan nanoparticles stable over a wide pH range by adsorption of polyglycerol monostearate. Nanomater. Nanotechnol..

[44] Won H.I., Nersisyan H., Won C.W., Lee J.-M., Hwang J.-S. (2010). Preparation of porous silver particles using ammonium formate and its formation mechanism. Chem. Eng. J..

[45] Bregoli C., Fiocchi J., Biffi C.A., Tuissi A. (2023). Additively manufactured medical bone screws: An initial study to investigate the impact of lattice-based Voronoi structure on implant primary stability. Rapid Prototyp..

[46] Renzo D.A., Sgambitterra E., Magarò P., Furgiuele F., Maletta C., Biffi C.A., Fiocchi J., Tuissi A. (2021). Multiaxial fatigue behavior of additively manufactured Ti6Al4V alloy: Axial–torsional proportional loads. Mater. Des. Process. Commun..

[47] Esfahani A.G., Altomare L., Varoni E.M., Bertoldi S., Farè S., De Nardo L. (2019). Electrophoretic bottom up design of chitosan patches for topical drug delivery. J. Mater. Sci. Mater. Med..

[48] Isfahani A.G., Ghorbani M. (2013). Electrophoretic Deposition of Ni/SiO_2_ Nanocomposite Coating: Fabrication Process and Tribological and Corrosion Properties. J. Nano Res..

[49] Ghalayani Esfahani A., Lazazzera B., Draghi L., Farè S., Chiesa R., De Nardo L., Billi F. (2018). Bactericidal activity of Gallium-doped chitosan coatings against staphylococcal infection. J. Appl. Microbiol..

[50] Ghalayani Esfahani A., Lazazzera B., Draghi L., Farè S., Chiesa R., De Nardo L., Billi F. (2020). Micro-Structured Patches for Dermal Regeneration Obtained via Electrophoretic Replica Deposition. Appl. Sci..

[51] Ghalayani Esfahani A., Oleimanzade M., Campiglio C.E., Federici A., Altomare L., Draghi L., Boccaccini A.R., De Nardo L. (2019). Hierarchical microchannel architecture in chitosan/bioactive glass scaffolds via electrophoretic deposition positive-replica. J. Biomed. Mater. Res. Part A.

[52] Brouwer J., van Leeuwen-Herberts T., de Ruit M.O. (1984). Determination of lysozyme in serum, urine, cerebrospinal fluid and feces by enzyme immunoassay. Clin. Chim. Acta.

[53] Porstmann B., Jung K., Schmechta H., Evers U., Pergande M., Porstmann T., Kramm H.J., Krause H. (1989). Measurement of lysozyme in human body fluids: Comparison of various enzyme immunoassay techniques and their diagnostic application. Clin. Biochem..

[54] (2009). Biological Evaluation of Medical Devices—Part 5: Tests for In Vitro Cytotoxicity.

[55] Cárdenas G., Miranda S.P. (2004). FTIR and TGA studies of chitosan composite films. J. Chil. Chem. Soc..

[56] Kittur F.S., Prashanth K.V.H., Sankar K.U., Tharanathan R.N. (2002). Characterization of chitin, chitosan and their carboxymethyl derivatives by differential scanning calorimetry. Carbohydr. Polym..

[57] Aimin C., Chunlin H., Juliang B., Tinyin Z., Zhichao D. (1999). Antibiotic Loaded Chitosan Bar: An In Vitro, In Vivo Study of a Possible Treatment for Osteomyelitis. Clin. Orthop. Relat. Res..

[58] Valour F., Trouillet-Assant S., Rasigade J.P., Lustig S., Chanard E., Meugnier H., Tigaud S., Vandenesch F., Etienne J., Ferry T. (2013). *Staphylococcus epidermidis* in Orthopedic Device Infections: The Role of Bacterial Internalization in Human Osteoblasts and Biofilm Formation. PLoS ONE.

[59] Huq A., Parvez A.K., Balusamy S.R. (2022). Chitosan-Coated Polymeric Silver and Gold Nanoparticles: Biosynthesis, Characterization and Potential Antibacterial Applications: A Review. Polymers.

[60] Lanzi O. (1990). Effect of Pore Structure on Current and Potential Distributions in a Porous Electrode. J. Electrochem. Soc..

[61] Zhitomirsky I., Gal-or L. (1997). Electrophoretic deposition of hydroxyapatite. J. Mater. Sci. Mater. Med..

[62] Pangburn S., Trescony P., Heller J. (1982). Lysozyme degradation of partially deacetylated chitin, its films and hydrogels. Biomaterials.

[63] Lee K.Y., Ha W.S., Park W.H. (1995). Blood compatibility and biodegradability of partially N-acylated chitosan derivatives. Biomaterials.

[64] Tomihata K., Ikada Y. (1997). In vitro and in vivo degradation of films of chitin and its deacetylated derivatives. Biomaterials.

[65] Vårum K.M., Myhr M.M., Hjerde R.J.N., Smidsrød O. (1997). In vitro degradation rates of partially N-acetylated chitosans in human serum. Carbohydr. Res..

[66] Nordtveit R.J., Vårum K.M., Smidsrød O. (1994). Degradation of fully water-soluble, partially N-acetylated chitosans with lysozyme. Carbohydr. Polym..

[67] Zilberman M., Elsner J.J. (2008). Antibiotic-eluting medical devices for various applications. J. Control. Release.

[68] Simchi A., Tamjid E., Pishbin F., Boccaccini A.R. (2011). Recent progress in inorganic and composite coatings with bactericidal capability for orthopaedic applications. Nanomedicine.

[69] Ordikhani F., Tamjid E., Simchi A. (2014). Characterization and antibacterial performance of electrodeposited chitosan-vancomycin composite coatings for prevention of implant-associated infections. Mater. Sci. Eng. C.

[70] Bernthal N.M., Stavrakis A.I., Billi F., Cho J.S., Kremen T.J., Simon S.I., Cheung A.L., Finerman G.A., Lieberman J.R., Adams J.S. (2010). A Mouse Model of Post-Arthroplasty *Staphylococcus aureus* Joint Infection to Evaluate In Vivo the Efficacy of Antimicrobial Implant Coatings. PLoS ONE.

[71] Zou X., Zhao X., Ye L., Wang Q., Li H. (2015). Preparation and drug release behavior of pH-responsive bovine serum albumin-loaded chitosan microspheres. J. Ind. Eng. Chem..

[72] Kim I.Y., Seo S.J., Moon H.S., Yoo M.K., Park I.Y., Kim B.C., Cho C.S. (2008). Chitosan and its derivatives for tissue engineering applications. Biotechnol. Adv..

[73] Polinarski M.A., Beal A.L., Silva F.E., Bernardi-Wenzel J., Burin G.R., de Muniz G.I., Alves H.J. (2021). New Perspectives of Using Chitosan, Silver, and Chitosan–Silver Nanoparticles against Multidrug-Resistant Bacteria. Part. Part. Syst. Charact..

